# Editorial: Case reports in autoimmune and autoinflammatory disorders

**DOI:** 10.3389/fimmu.2025.1655791

**Published:** 2025-08-22

**Authors:** Chris Wincup, Augusto Vaglio, Stefano Volpi, Gian Marco Ghiggeri

**Affiliations:** ^1^ Department of Clinical and Academic Rheumatology, King’s College Hospital, London, United Kingdom; ^2^ Nephrologyand Dialysis Unit, Meyer Children’s Hospital IRCCS, Florence, Italy; ^3^ Department of Biomedical, Experimental and Clinical Sciences “Mario Serio”, University of Florence, Florence, Italy; ^4^ Rheumatology, Institute Giannina Gaslini, Genoa, Italy; ^5^ Nephrology, Institute Giannina Gaslini, Genoa, Italy

**Keywords:** autoimmune disorders, inflammatory diseases, laboratory biomarkers, case reports, clinical features

Autoimmune and autoinflammatory disorders include a diverse group of multi-system clinical conditions that are characterized by either autoimmunity or exaggerated and persistent inflammatory responses as their basic mechanisms. In recent years, research investigating the basic science, immunology and translational medicine has played a crucial role in expanding our knowledge of the mechanisms involved in the pathogenesis of autoimmune diseases. Technologies for detecting proteins and small molecules, and the development of system analysis for large amounts of data (enhanced by advances in machine learning and artificial intelligence) have played a central role in identifying new auto-antibodies and mediators of inflammation while further contributing to the definition of the new borders of this evolving field. The availability of new drugs, particularly targeted monoclonal antibodies, immunotherapies and cell-based therapies, has also transformed the management of patients who for decades have experienced the effects of less targeted therapies, such as long-term steroids and anti-inflammatory agents. Given that many of these conditions are both rare and heterogeneous in terms of clinical manifestations and immunological drivers, many large clinical trials have sadly failed to achieve the primary endpoint. However, data from case reports, case series and open-label studies have seen many of these agents adopted for use in clinical practice, particularly in severe, refractory, or atypical cases.

Single case reports contain many elements that may help clinicians to resolve medical conditions in the setting of either challenging or difficult solutions, and represent a reasonable approach that may impact research through the generation of new data that may prompt further future investigation. Publishing case reports also involves an editorial effort associated with the risk that a new finding will not be directly linked to the disease pathogenesis but will represent an epiphenomenon that is useless to report. However, the simple observation of well-reported cases may provide a solid basis for future discoveries and stimulate further discussion in the field (particularly in rare or atypical cases).

The Research Topicwas not limited to simple descriptions of single cases, but was also open to either systematic reviews of the literature or original research describing proteomics laboratory platforms that focus on exceptional, severe or rare conditions. This first volume of this Research Topic of Frontiers of Immunology was characterized by a wide variety of topics covered, the strong scientific basis of individual reports, and, in those cases describing new therapeutic approaches, the possibility that they may significantly impact clinical medicine.

We commend the reviewers for their efforts in evaluating the quality and interest of the manuscripts submitted for this special edition. Interest in this area was high with 150 submissions in total, nearly half of which were accepted for publication in this Research Topic; this is in line with the general acceptance of the journal and highlights the rigorous evaluation process that the reviewers employed for all submissions.

## Areas covered by volume I

Interestingly, three areas of medicine (Neurology, Dermatology and Rheumatology) accounted for 85% of the published papers, which is in line with the growing innovations that these areas are witnessing. Neurology was the most popular topic (39% of accepted papers), followed by Dermatology (24%) and Rheumatology (22%). Other important areas, such as Hemato-Oncology, Nephrology, Gastroenterology, and Ocular diseases, accounted for 15% of published cases.

When considering specific disorders, encephalitis was the main neurological topic in this Research Topic. This included anti-N-methyl-D-aspartate receptor (NMDAR) encephalitis presenting with atypical features (e.g., psychosis, cerebellar symptoms) and its treatments such as the chimeric anti-CD20 monoclonal antibody-Rituximab) [ (1), Reda et al., Zhang et al., Xu et al.)], anti-Purkinje antibodies in PCA2 encephalitis (Li et al.), and one case of seronegative limbic encephalitis (Los et al.). The treatment of myasthenia gravis with Telitacicept (Zhang et al.) and of autoimmune necrotizing myopathy with the humanized anti-CD20 monoclonal antibody Ofatumumab (Chen et al.) highlights the role of targeting B cells in these diseases and may indicate this as an interesting area for new therapies. Furthermore, the role of TNFα inhibitors (TNF-αi) in neurological disorders was also discussed in one paper in this Research Topic (Kassabian et al.).

Several neurological cases with atypical clinical presentations that required novel laboratory approaches to reach a correct diagnosis were also submitted. Of note, one case involved a patient with Guillain-Barrè syndrome who presented with paralytic ileus. The diagnosis of Guillen-Barrè was reached based on autoimmune tests for anti-sulfatide antibodies, anti-GD1a antibodies, and anti-GT1a antibodies. Based on these results, the patient was treated and rapidly improved after plasma exchange and intravenous immunoglobulin treatment, thus avoiding significant bowel surgery (2). Neuropathic pain as the unique sign of nerve hyperexcitability occurring in the context of autoimmune targeting of the potassium channel complex was described in a young woman presenting with high serum levels of anti-contactin-associated protein-like 2 (Zhu et al.). This case highlights that complete resolution of this specific type of neuropathic pain can be brought about through the use of corticosteroids and immunoglobulin. This case also highlights how pain may be the first clinical sign of a severe pathology that is difficult to treat in advanced stages.

The major focus of the Dermatology collection of articles in this Research Topic was the use of innovative immunotherapies in cutaneous diseases. These included the use of Spesolimab and Secukinumab for psoriasis and Hallopeau acrodermatitis (Wen et al.), the use of Baricitinib with Dupilumab for severe alopecia associated with atopic dermatitis (Fang et al.), the role of Etanercept in necrotic epidermolysis (Jeong et al.), and the first case of perforating collagenosis treated with Baricitinib (Zheng et al.). Adverse events were also reported in a case of dermatomyositis induced by Imatinib (Silva et al.) an antineoplastic drug and tyrosine kinase inhibitor primarily used in the treatment of leukemia and stromal gastrointestinal cancer.

Rheumatology-focused case reports were predominantly descriptive of rare associations, such as VEXAS syndrome (Vacuoles, enzyme E1, X-linked, autoinflammatory, somatic) (Diral et al., Costa et al.), antiphospholipid syndrome (Chicharo et al.), and a case of lethal IL-1 deficiency supported by IL1-rec mutations (Urbaneja et al.). VEXAS syndrome is an acquired autoinflammatory disease that is characterized in the majority of cases by myelodysplastic disorders accompanied by cytopenia, macrocytic anemia, fever, skin vasculitis, and pleuropulmonary disorders. It is believed to be caused by somatic mutations in the UBA1 gene, which lead to the aberrant activation of the innate immune system and the production of proinflammatory cytokines. This Special Issue included descriptions of seven cases of VEXAS syndrome classified according to the 2022 WHO guidelines based on morphological, cytogenetic and molecular characteristics, which may help in proper treatment (Diral et al.). Another reported VEXAS case was a patient who presented atypically with sacroiliitis as an indolent symptom that responded to azacitidine (Costa et al.).

Several reports were submitted on antiphospholipid syndrome, including its association with COVID-19 infection (Li et al.). A case of complex tachycardia with atrioventricular block plus Wolf Parkinson White syndrome (WPW) transmitted to her fetus by a mother positive for anti-SSA antibodies supports the importance of a clear medical characterization before pregnancy in patients with a potential autoimmune condition (Chicharo et al.). Furthermore, a case of rheumatoid arthritis treated with Etanercept who developed aseptic meningitis was reported, indicating that this drug may cross the blood-brain barrier and exert toxicity. This highlights the challenges faced clinically with infections, which are a well-known risk of immunosuppressive therapy (Jeong et al.).

Additional cases included in this volume were the first description of DRESS syndrome following the use of sulfasalazine to treat COVID-19 (Li et al.), and the successful treatment of Evans syndrome (hemolytic anemia with thrombocytopenia) with JAK inhibition (3). The potential occurrence of renal complications following immunotherapy with humanized monoclonal antibody against the programmed death cell protein (anti-PD1), which is now more frequently being used in the treatment of malignancy, was discussed in a paper reporting a case of IgA glomerulonephritis following pembrolizumab in a patient with non-small cell lung carcinoma (Chabannes et al.). Recognition of the adverse effects of anti-PD1 therapy is very important given the rapid increase in the use of these checkpoint inhibitors (Yang et al.) for different cancer types in which these antibodies are utilized (including melanoma and cancers of major organs such as the liver, lungs, colon, and others).

Autoimmune gastritis is a form of atrophic gastritis that may evolve to gastric neuroendocrine cancer and should be recognized and treated in the early stages. Diffuse homogeneous atrophy of the gastric body is a major endoscopic characteristic of advanced cases, although it is not always present at the onset of the disease. The endoscopic characteristics of two cases of early autoimmune gastritis were described and discussed in this Research Topic. The presence of anti-parietal cell antibodies confirmed the diagnosis in both cases, allowing for direct therapy for the autoimmune condition (Yu et al.).

The Research Topicalso contains two papers that can be considered atypical for a ‘Case report collection’. One of these is a systematic review of fifty-three cases of pemphigus associated with end-stage renal failure, in addition to including eight relevant studies on the topic (Yang et al.). The efficacy of major therapies based on immunosuppression was widely discussed. The second paper was an original research study describing a proteomic laboratory platform that focused on patients with exceptionally severe Myalgic Encephalomyelitis/Chronic Fatigue Syndrome (ME/CFS) and hypermobility spectrum disorders (Jahanbani et al.). The correct diagnosis of ME/CFS was made by profiling the Th2 cytokine levels, which highlighted a synergistic relationship between mast cells and eosinophils – a finding of interest for potential future biomarker investigations.

## Proposals for volume II

Following the success of Volume I, Volume II proposes to expand on the same topics and serve as a continuation of Volume I. Descriptions of single cases including those in the context of an accompanying review of the literature continue to be encouraged for submission. In addition, systematic reviews of literature on rare conditions are welcome. Original research will be considered if reporting and validating new laboratory assays for identifying biomarkers, such as auto-antibodies and cytokines that either facilitate the diagnosis or potentially anticipate clinical outcomes of autoimmune or auto-inflammatory conditions.

We welcome manuscripts focusing on, but not limited to, the following areas:

- Description of new links between autoimmunity and diseases of any kind
- Identification of new autoantibodies
- Identification of immune mechanisms involved in inflammation.
- Characterization of immune biomarkers of clinical worsening
- Factors predicting relapses and non-response to therapies
- New therapeutic targets and description of ‘side effects’ with an emphasis on immunotherapies

Therapies will have a special space in Volume II and, in consideration of the incredible evolution that we are witnessing in the area of ‘immunotherapies’, we hope that Volume II will attract the interest of clinicians involved in testing new human monoclonal antibodies that target specific immune cells (e.g., anti-CD20, anti-CD38 and others) along with cytokines, JAK inhibitors and anti-PD1/PD-1L in cancer. There is a vast area of autoimmune conditions that could potentially respond to newer monoclonal antibodies that is still to be investigated and now represents the new frontier.

Case-control studies of small to medium size also represent an important area in contrast to single cases, and we appreciate studies that present the positive effects of new treatments which could form the basis for important trials in the future. Descriptions of the adverse effects of new therapies are also welcome if they indicate a direct relationship, such as a rapid onset after the start of a therapy or a cessation of symptoms after withdrawal.

Volume I of this Research Topichas outlined that there is still much to learn in the identification and management of autoimmune and autoinflammatory diseases, while effectively demonstrating the value of well-written and highly innovative case reports in supplementing the existing literature.
